# Methylation Dynamics of *RASSF1A* and Its Impact on Cancer

**DOI:** 10.3390/cancers11070959

**Published:** 2019-07-09

**Authors:** Giorgio Malpeli, Giulio Innamorati, Ilaria Decimo, Maria Bencivenga, Armel Herve Nwabo Kamdje, Roberto Perris, Claudio Bassi

**Affiliations:** 1 Department of Surgical Sciences, Dentistry, Gynecology and Pediatrics, Section of Surgery, University of Verona, 37134 Verona, Italy; 2 Department of Diagnostics and Public Health, Section of Anatomic Pathology, University of Verona, 37134 Verona, Italy; 3 Department of Medicine, Section of Pharmacology, University of Verona, 37134 Verona, Italy; 4 Department of Biomedical Sciences, University of Ngaoundere, Ngaoundere 454, Cameroon; 5 Department of Biosciences, COMT-Centre for Molecular and Translational Oncology, University of Parma, 43124 Parma, Italy

**Keywords:** *RASSF1*, *RASSF1A*, DNA methylation, grastrointestinal cancers, biomarker

## Abstract

5-methyl cytosine (5mC) is a key epigenetic mark entwined with gene expression and the specification of cellular phenotypes. Its distribution around gene promoters sets a barrier for transcriptional enhancers or inhibitor proteins binding to their target sequences. As a result, an additional level of regulation is added to the signals that organize the access to the chromatin and its structural components. The tumor suppressor gene *RASSF1A* is a microtubule-associated and multitasking scaffold protein communicating with the RAS pathway, estrogen receptor signaling, and Hippo pathway. *RASSF1A* action stimulates mitotic arrest, DNA repair and apoptosis, and controls the cell cycle and cell migration. De novo methylation of the *RASSF1A* promoter has received much attention due to its increased frequency in most cancer types. *RASSF1A* methylation is preceded by histones modifications and could represent an early molecular event in cell transformation. Accordingly, *RASSF1A* methylation is proposed as an epigenetic candidate marker in many cancer types, even though an inverse correlation of methylation and expression remains to be fully ascertained. Some findings indicate that the epigenetic abrogation of *RASSF1A* can promote the alternative expression of the putative oncogenic isoform *RASSF1C*. Understanding the complexity and significance of *RASSF1A* methylation is instrumental for a more accurate determination of its biological and clinical role. The review covers the molecular events implicated in *RASSF1A* methylation and gene silencing and provides a deeper view into the significance of the *RASSF1A* methylation patterns in a number of gastrointestinal cancer types.

## 1. The Tumor Suppressor *RASSF1A*

**Figure 1:**
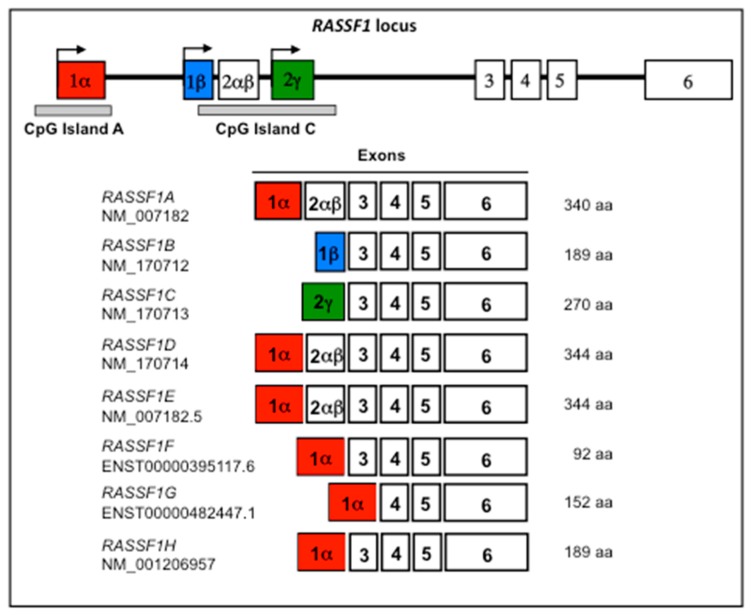
Schematic representation of the *RASSF1* locus and its transcription map. White boxes represent the exons and the bold line represents the introns. *RASSF1A* (red), *RASSF1B* (blue), and *RASSF1C* (green) variants are generated by differential promoter and first exon usage (arrows). *RASSF1D*, *RASSF1E*, *RASSF1F*, *RASSF1G*, and *RASSF1H* are variants derived from alternative splicing of *RASSF1A*. Two CpG islands (grey bands below exons 1 and 2) are associated to *RASSF1* promoter region: CpG island A (736 bp, 85 CpGs, chr3:50,340,373-50,341,109, GRCh38/hg38) extending in the promoter region of *RASSF1A;* CpG island C (1364 bp, 139 CpGs, chr3:50,336,834-50,338,198, GRCh38/hg38) in the regulatory region of *RASSF1B* and *RASSF1C*. A CpG island is defined as a sequence with a length greater than 200 bp, a GC content greater than 50% and a ratio greater than 0.6 of the observed number of CG dinucleotides with respect to the expected number on the basis of the number of Gs and Cs nucleotides in the segment.

The *RASSF1* locus, in the cytogenetic band chr3:p21.31, expresses eight main transcript variants under the control of two promoters overlapped to the CpG islands A and C [1]. The major, ubiquitous transcripts expressed by the *RASSF1* locus are *RASSF1A* and *RASSF1C* [1,2]. *RASSF1B* and *RASSF1C* differ in the first exon. Additional *RASSF1* isoforms, *RASSF1D*, *RASSF1E*, *RASSF1F*, *RASSF1G*, and *RASSF1H* derive from alternative splicing of *RASSF1A*. A schematic representation of the *RASSF1* locus is reported in Figure 1.

The first exon of *RASSF1A* contains a cysteine-rich domain, reminiscent of the diacylglycerol-binding–CRD domain [1]. This domain is lacking in *RASSF1B* and *RASSF1C*. Most studies published on *RASSF1* concern *RASSF1A* and *RASSF1C*. The function of *RASSF1B*, *RASSF1D*, *RASSF1E*, *RASSF1F*, *RASSF1G*, and *RASSF1H* has received little research attention.

The *RASSF1A* isoform is transcribed in the *RASSF1* locus about 180 base pairs aside to the gene *ZMYND10* (protein name BLU). Three binding sites for the insulator CCCTC-binding factor (*CTCF*) are overlapped to the 3′ end of *ZMYND10*, in a region between −453 and −2050 bp from the *RASSF1A* transcription start site. The insulator CTCF binds insulator sequences that separate functionally the transcription start sites of the two close genes forming two distinct epigenetic domains [3]. For this reason, despite their proximity, *RASSF1A* has often been found down-regulated in cancer as opposed to *ZMYND10* [4].

The 3p.21.31 region may harbor candidate tumor suppressor genes due to the frequent copy number loss in various cancer types [5,6,7], including *HYAL2*, *TUSC2*, *RASSF1*, *ZMYND10*, *NPRL2*, *CYB561D2*, *TMEM115*. and *CACNA2D2* [8]. The gene *RASSF1A* (*RASSF1*-association domain family 1, isoform A) was initially identified as a potential RAS binding molecule due to the presence of a RAS-association domain in its primary sequence. *RASSF1A* binds RAS in the GTP-bound form to promote apoptosis [9]. *RASSF1A* has been proposed to act as a tumor suppressor, since the loss of its function induces cell proliferation and tumorigenesis [10], and may be implicated in microtubule stabilization, apoptosis, cell cycle, and DNA repair [11,12,13,14,15]. The most relevant cell protection functions operated by *RASSF1A* are mediated by the interaction with the Hippo and the Wnt pathways and the modulator of apoptosis 1 (MOAP1) [16,17,18,19,20,21]. Aged *RASSF1A*(−/−) mice are prone to spontaneous tumorigenesis [20], particularly in the liver [22], suggesting that *RASSF1A* function is essential for a correct homeostasis and healthy state of cells. Moreover, DNA methylation and silencing of *RASSF1A*, along with another tumor suppressor gene, HIC1, transforms mesenchymal stem cells into cancer stem cells [23], implying that *RASSF1A* methylation (*RASSF1A*m) is part of a delicate hierarchical network of oncogenic gene silencing patterns involved in neoplastic transformation. In accordance with the above mentioned observations, *RASSF1A* is expressed in all normal tissues and at a lower level than *RASSF1C* [24]. Reverting *RASSF1A* down-regulation in cancer cell lines restores controlled growth and colony formation, as well as decreased cell migration and apoptosis [10,25,26,27,28,29,30].

## 2. Biological Role of *RASSF1C*

The function of the isoform *RASSF1C* is less well defined with respect to that of *RASSF1A*, although various studies converge upon the idea that the molecule could promote cell survival and proliferation, to thereby exert an opposite role to that attributed to *RASSF1A*. *RASSF1C* has been demonstrated to be nuclear or perinuclear with translocation to the cytosol upon DNA damage [31], or localized to microtubules similarly to *RASSF1A* [32,33]. The Daxx-*RASSF1C* complex has been shown to be involved in the DNA damage response and the SAPK/JNK signaling pathway [34]. Initially, *RASSF1C* has been shown to induce cell cycle arrest in cancer cell lines suggesting that, like *RASSF1A*, the *RASSF1C* gene could act as a tumor suppressor function [35]. Other findings support a potential role of *RASSF1C* as oncogene, promoting beta-catenin (*CTNNB1*) accumulation in HeLa cells [36] and proliferation of lung cancer cells [37], supporting cell migration and attenuated apoptosis in breast cancer [38].

## 3. DNA Methylation Changes

Methylation of cytosine to 5-methylcytosine (5mC) is a central epigenetic modification that feeds back on cellular processes including genome regulation organism development and disease. DNA methyltransferases DNMT1, DNMT3a, and DNMT3b establish specific 5mC patterns during embryonic development and cell differentiation and maintain them over many cell division cycles in adults [39]. DNMT1 is the enzyme responsible for the addition of methyl groups, immediately following DNA replication, preferentially to hemimethylated DNA. DNMT1 is post-transcriptionally regulated by a mutually exclusive Ser/Thr phosphorylation and Lys methylation under the control of PI3K-AKT-mTOR pathway [40,41,42]. DNMT3a and DNMT3b are preferentially implicated in de novo DNA methylation, that is the addition of one methyl group to cytosine in unmethylated CpG dinucleotides after DNA duplication [43]. DNA methylation can be reversed by Ten-eleven translocation (TET) enzymes (*TET1*, *TET2*, *TET3*), which are responsible for the fine-tuning methylation of patterns [44]. TET enzymes oxidize the methyl group of 5mC to yield 5-hydroxymethylcytosine, which facilitates both passive and active demethylation. The implications of methylation-demethylation epigenetic disequilibrium and of TET enzymes in gastrointestinal cancers have been observed in various studies [45,46,47,48,49,50,51]. Understanding DNA methylation-demethylation dynamics, and their epigenetic interplays in modulating transcription will open new perspectives for research on cellular differentiation and oncogenic transformation (see Ambrosi et al. [52] for a review).

DNMT1, DNMT3A, and DNMT3B enzymes are responsible for RASSF1Am in different contexts [53,54,55,56,57,58]. RASSF1Am seems to follow a precise cascade of events with recruitment of the complex HDAC1/SETDB1, that in turn attracts DNMT3A in cancer cells [58]. In lung cancer, ΔDNMT3B4 (DNMT3 that lacks exon 6) appears essential for *RASSF1A* silencing [55], while its high methylation profile is driven by other epigenetic signals to support gene silencing architecture that favors cancer growth [59].

5mCs are recognized by methyl-binding proteins that in turn recruit histone modifying and chromatin remodeling enzymes [60]. Capped 5mCs promote a closed chromatin structure by obstructing the binding of transcription factors (inhibitors and enhancers of gene transcription) [61,62]. Somatic reprogramming is a dramatic demonstration of the impact of DNA methylation on cell fate [63]. 5mCs are less frequent in the target sequences of transcription factors, or are selectively over-represented in some CpG islands, but are yet influencing transcriptional programs [64], suggesting that fine-tuned DNA methylation tends to be dynamically and functionally interconnected with cellular signaling pathways [65] (see Du et al. [66] for a review).

## 4. Methylation of RASSF1A in Normal Tissues of the Gastroenteric System

**Figure 2:**
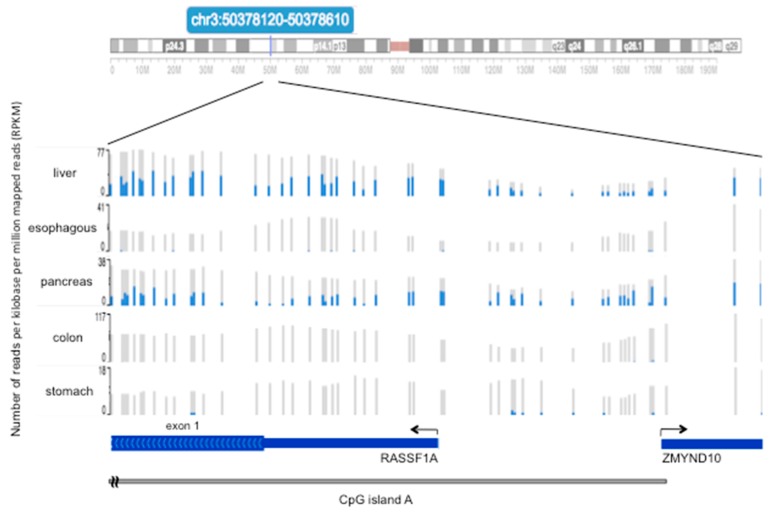
Methylation level of 48 CpGs in the promoter and first exon of *RASSF1A* in five normal tissue types. Results of bisulfite DNA sequencing on liver, esophagus, pancreas, colon, and stomach tissues samples taken from a 34-year-old healthy subject. The grey/blue vertical bars represent 48 CpGs overlapped to the promoter of *RASSF1A* and partially to first exon and CpG island A. For each CpG, the blue line is the number of reads for the 5mC and grey is the total number of reads (number of reads per kilobase per million mapped reads, RPKM) obtained by bisulfite DNA sequencing. The ratio between the two numbers represents the methylation level of a CpG. The grey bar at the bottom of the figure identifies the 3′ end of the CpG island A. The data were retrieved by the WashUp EpiGenome Browser at www.epigenomegateway.wustl.edu, provided by the Roadmap EpiGenome Project [69].

*RASSF1A*m usually refers to the methylation of the CpG island A, which covers the promoter and first exon of *RASSF1A*. There are few descriptions of the methylation status of *RASSF1A* at single CpG resolution in normal gastrointestinal tissues. Heterogeneous distribution and level of 5mCs in adjacent CpGs in the *RASSF1A* promoter and first exon was observed in the normal pancreas adjacent to pancreatic endocrine tumors [67]. In these individuals, CpG methylation ranged from absent to diffuse. Figure 2 shows the variable CpG methylation patterns found in five normal tissues of a 34-year-old healthy individual. In this example, CpG methylation was higher in the liver and pancreas and was almost absent in the esophagus, colon and, stomach. According to data reported in Figure 2, unmethylated *RASSF1A* status was found in 15 stomach normal tissues [68]. The Cancer Genome Atlas (TCGA) data confirmed a variable and higher *RASSF1A*m level in the normal liver and pancreas compared to colon and stomach (see Section 7).

## 5. Mechanisms of *RASSF1A* Methylation in Cancer and Aging

All cancers are characterized by some degree of global epigenetic alteration entailing general DNA hypomethylation and abnormal hypermethylation in specific CpG islands. Alteration of DNA methylation patterns may depend on altered methyl group transfer during DNA duplication, or on defects of 5mC hydroxylation and demethylation operated by TET enzymes. In cancer and during aging, a substantial fraction of genes undergo a cell type-specific DNA hypermethylation of silenced genomic loci protein that is preceded by H3-K27 and H3-K9 trimethylations [70,71,72,73,74,75,76]. Analogously, an aberrant transcriptional silencing of *RASSF1A* triggered by the inactivating chromatin modification histone deacetylation and H3-K9 methylation preceded CpG island A hypermethylation [59] (see Klutstein et al. [77] for a review).

In differentiated cells, CpG islands particularly rich in CpGs and overlapped to gene promoters, as those present in *RASSF1A*, remain mostly unmethylated, even when the gene is inactive [59,78,79,80]. Thus, the occurrence of 5mCs in the CpG island A (Figure 2) could be considered as a somatically acquired abnormal event that spreads through the core region to initiate gene silencing, possibly reflecting distinct interactions among epigenetic machinery and components of the chromatin responsible for transcriptional regulation. [75,81,82].

*RASSF1A* is hypermethylated in most cancer types, and in some cases also in the adjacent normal tissues [1,83]. Aberrant *RASSF1A* promoter DNA methylation has been detected also in childhood neoplasia, including neuroblastoma, thyroid carcinoma, hepatocellular carcinoma, pancreatoblastoma, adrenocortical carcinoma, Wilms’ tumor, Burkitt’s lymphoma, and T-cell lymphoma [78]. The methylation of CpGs in the promoter and first exon of *RASSF1A* shows extreme variability in terms of distribution and relative levels of each CpG in single cell types, as well as in cancer and normal tissues [10,27,67,68,84,85,86,87,88,89,90]. This may possibly reflect a cell type- and/or clonal-based epigenetic heterogeneity.

It is reported that internal or environmental stimuli can promote epigenetic modifications that spread as silent events [85]. For example, *RASSF1A*m increases during physiological or patho-physiological processes such as aging, hypoxic conditions, senescence, inflammation, and viral infection [30,75,77,91,92,93,94,95]. De novo *RASSF1A*m associates with different factors and conditions; folate metabolism, DNA polymorphisms, as well as choline-deficient L-amino acid-defined diet in rats [96,97]. Early stages of estrogen-induced breast carcinogenesis in female rats is characterized by altered global DNA methylation, aberrant expression of proteins responsible for maintenance of DNA methylation pattern, and also by de novo *RASSF1A*m [98]. Transfection of hepatitis C virus core protein into hilar cholangiocarcinoma cell lines induces *RASSF1A* promoter DNA methylation and silencing [95]. In these contexts, *RASSF1A*m would function as an epigenetic sensor, associated to physiologic and disease conditions. The cell progeny could inherit a gene dosage pernicious for the *RASSF1A*-dependent cell functions that remains altered for the entire life of the organism. According to this model, de novo DNA methylation or demethylation at regulatory sites can anticipate the pathological transformation in different cell phenotypes before transformation ensues (epigenetic field defect) [85,99,100].

An age-dependent increase of *RASSF1A*m at differing speeds in different organs of healthy individuals is largely recapitulated in corresponding cancer types [74,75,76,77]. Thus, site-specific DNA hypermethylations that overlap in aging and tumorigenesis candidate these sites as cancer susceptibility hotspots. The epigenetic changes and the parallel increased risk of tumor onset occurring during aging keep open the possibility of a causative role of epigenetic reprogramming in *RASSF1A* silencing in support of progressive tumorigenesis.

## 6. Relationship between *RASSF1A* Methylation and Expression

The mechanisms that regulate DNA methylation and its consequences on gene transcription are only partially understood. The relative levels and patterns of methylation at specific CpG sites along the entire genome associate variably with gene expression [101]. More precisely, for each gene the correlation can be both positive or negative in different cell types suggesting tissue-specificity [102]. However, data relative to *RASSF1* derived from omics techniques should be considered with caution since the applied experimental procedures might have not been sufficiently sensitive and able to provide high resolution data in the *RASSF1* genomic region. *RASSF1A* expression is lost in different cancer types, as in lung, breast, and kidney cancer [103,104]. Consistently, epigenetic loss of *RASSF1A* has been proposed to serve as a diagnostic marker of clinical outcome in some cancer types [90,100,105,106,107].

There is currently poor understanding of the functional relevance of methylation of single CpG. From a mechanistic point of view, the methylation of cytosines in the normally unmethylated CpG island A can determine inhibitory cumulative effects on *RASSF1A* transcription due to the action of methyl-binding proteins. In experiments based on reporter constructs containing an artificial *RASSF1A* promoter with four groups of four CpGs at increasing distance from the transcription start site, two separated clusters of four consecutive methylated CpGs (not other combinations) determined a 63% decrease in promoter activity. Oct1 and Sp1 transcription factors bound preferentially to regulatory sequences overlapped to the regulatory CpGs when unmethylated [108]. Volodko et al. screened CpGs methylation and searched for correlation with *RASSF1A* transcription in various cancer types [109]. In colorectal cancer, seven CpGs hotspot in the *RASSF1A* promoter have been described to contribute to most of the DNA methylation. In breast and thyroid cancers, the methylation level of single CpGs mirrors the average value for the whole promoter. In normal breast tissue, *RASSF1A* exon 1 is found methylated without affecting gene expression [87], whereas matched breast cancers tissues show *RASSF1A* hypermethylation in both exon 1 and spreading towards the promoter region in association with the gene silencing.

In pancreatic endocrine tumors (PET), it has been demonstrated that a down-modulation of *RASSF1A* correlates with increased methylation of 51 CpG in the CpG island A and *RASSF1C* expression [67]. An expression switch between *RASSF1A* and *RASSF1C* concomitant to CpG island A hypermethylation has also been observed in breast cancer, neuroblastoma, some lung cancers [24,31], esophageal squamous cell carcinoma [110], renal cell carcinoma [111], breast, thyroid, and colorectal cancers [109], but not in pancreatic ductal adenocarcinoma [84]. *RASSF1A* is a Hippo pathway scaffold protein that subtracts YAP1 from oncogenic TEAD (TEA domain) transcriptional complexes and promotes tumor-suppressive YAP1/p73 activity [112]. *RASSF1A*m and alternated *RASSF1A* and *RASSF1C* expression correlates with loss of inhibitor signals mediated by YAP1, E-cadherin internalization and epithelial integrity is associated with an acquired invasive phenotype [113].

The alternated expression of distinct *RASSF1* isoforms with opposing functions would explain the association between loss of *RASSF1A* expression and an adverse outcome and disease progression for certain cancer types, calling upon the need of more functional studies to better understand the functional consequence of this switch. Furthermore, these data suggest that cell type-specific factors to be discovered modulate the transcriptional silencing of *RASSF1A* supported by the cytosine methylation.

## 7. *RASSF1A* Methylation and Expression in Gastrointestinal Cancers

By considering the published *RASSF1A*m data in gastrointestinal cancer types, the overall frequency of *RASSF1A*m is 78% in hepatocellular carcinoma, 34.6% in hepatoblastoma, 50% in esophageal squamous cell carcinoma, 54% in pancreatic ductal adenocarcinoma, 75% in PET, 35.6% in CRC and 31% in gastric cancer (Table 1).

**Figure 3:**
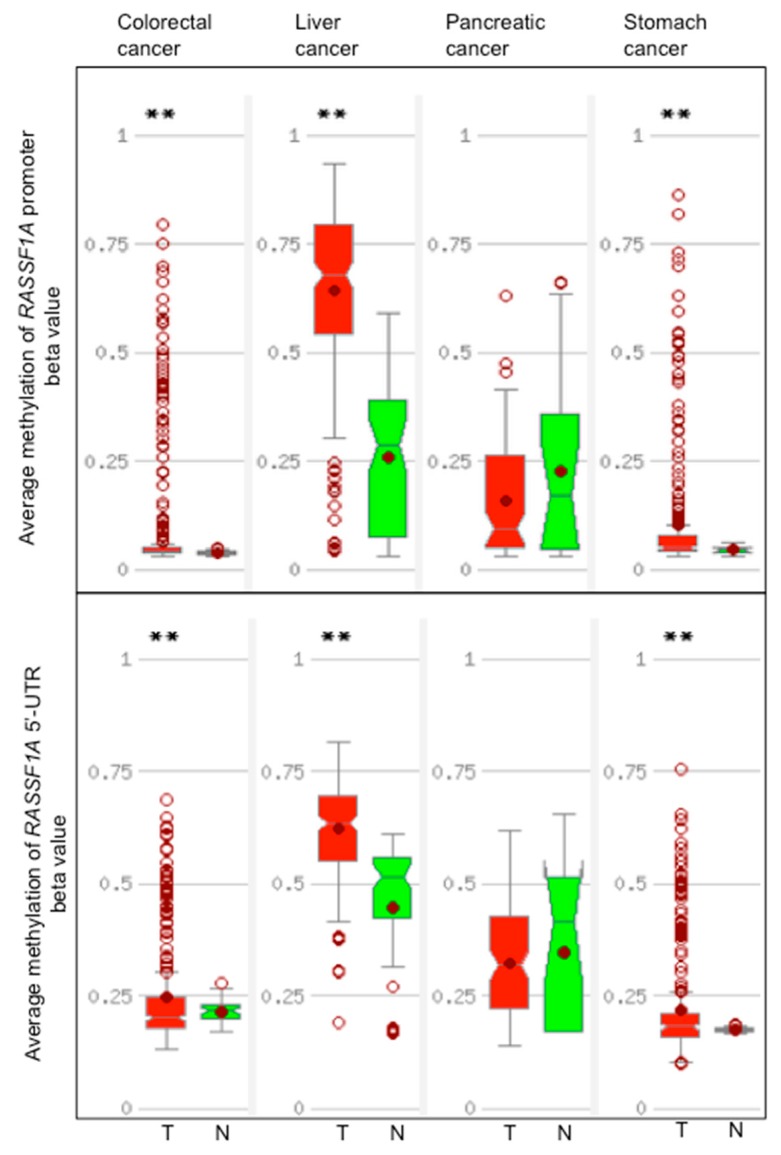
DNA methylation of the promoter and 5′-UTR of *RASSF1A* in colorectal, liver, pancreatic, and stomach cancers and in the corresponding normal tissues. Distribution of average methylation in the promoter and 5′-UTR of *RASSF1A* (NM_007182) in colorectal, liver, pancreatic, and stomach cancers cases. T, tumor tissues (red), N, normal tissue (green). 5′-UTR is the 5′ untranslated region of the first exon of *RASSF1A*. The promoter region is defined as from 1.5 kb upstream to 0.5 kb downstream of the *RASSF1A* transcription start site. The graphs show the distribution of beta values calculated as methylated probe intensity divided the unmethylated probe intensity plus methylated probe intensity, plus 100. In the boxes, a horizontal line represents the median and the filled circle the average; the box represents the 25th percentile interval and the whiskers the 95th percentile interval of the distribution. Empty points represent outliers. The double asterisk indicates a significant difference between T and N samples (*p* < 0.005) calculated by the *t*-test. The graphs, retrieved from the MethHC site [114], are based on The Cancer Genome Atlas (TCGA) data.

Data from TCGA show that *RASSF1A*m is detected at higher frequency as compared to normal tissues in liver, colorectal, and stomach cancers but not in pancreatic cancers (Figure 3).

The *RASSF1A*m data extracted from the literature and TCGA are consistent for liver, but not for pancreatic, colorectal, and gastric cancers. For pancreatic cancer, 20% to 35% *RASSF1A*m shown by TCGA data is close to the 35% reported by Amato et al. [84]. In general, differences of *RASSF1A*m may depend from the assay types applied and assay location, as discussed earlier in this review. A detailed description of the results and methods applied for the detection *RASSF1A*m in five gastrointestinal cancer types and the corresponding normal tissues is reported in Tables S1 to S5. All TCGA data were obtained by Illumina platforms. However, most of *RASSF1A*m data described in published studies were obtained by methylation-specific PCR (MSP), a qualitative technique informative of one or few CpGs. MSP tends to overestimate the frequency of DNA methylation as even few methylated CpGs belonging to a small fractions of the genomes present in the sample will produce a positive signal [115]. Given these premises, a certain variability in the association between DNA methylation and expression of *RASSF1A* or between *RASSF1A*m and the patients’ clinico-pathological parameters is expected to be found.

*RASSF1A* hypermethylation is a common finding in all gastrointestinal cancer types often along with other tumor suppressor genes in a pattern that is typical of CpG island methylator phenotype (CIMP). CIMP is an epigenetic disorder, characterized by widespread and simultaneous hypermethylation of CpG islands, that differentiates distinct subsets of cancer patients [116]. Genome-scale analysis found CIMP state generally concordant between primary colorectal cancers (CRCs) and corresponding metastases [117]. *RASSF1A* is candidate gene of CIMP in colorectal cancers [118] (see Weisenberger et al. [119] for e review).

**Figure 4:**
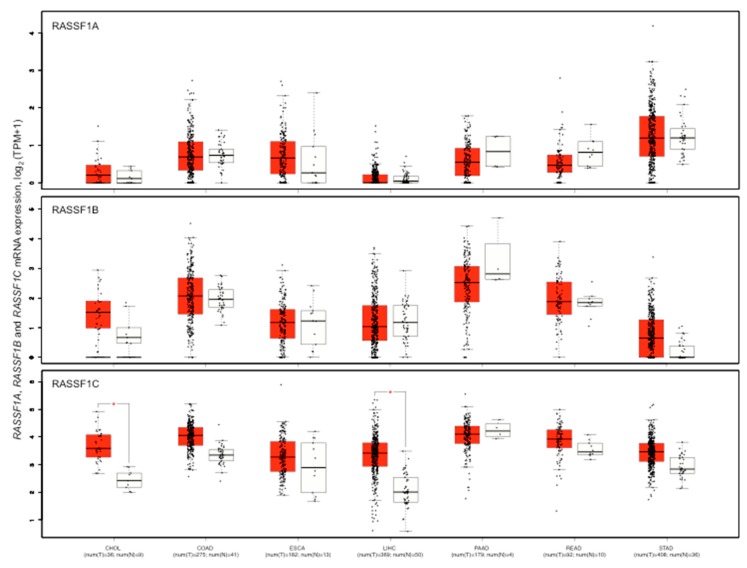
mRNA level of *RASSF1A*, *RASSF1B*, *RASSF1C* in seven gastrointestinal cancer types and the corresponding normal tissues. Red box, cancer types; white box normal tissues. The box plots show the expression distribution of the *RASSF1* isoforms *RASSF1A*, *RASSF1B*, and *RASSF1C* in the cancer types indicated in figure and the respective normal tissues. The expression levels of the samples are represented by black points. The expression data are log_2_ of Transcripts Per Million plus 1 (TPM+1) transformed for differential analysis and the log_2_ fold change defined as median of cancers minus median of normal tissues. Expression data of cancer and normal samples derived from the TCGA. Box plot represents the 25th percentile interval and whiskers represent the 95th percentile interval of the distribution. A horizontal line indicates the median value of the expressions. The number of samples for each series in cancers (T) and normal (N) is indicated below the acronym of cancer. CHOL, cholangiocarcinoma; COAD, colon adenocarcinoma; ESCA, esophageal carcinoma; LIHC, liver hepatocellular carcinoma; PAAD, pancreatic adenocarcinoma; READ, rectum adenocarcinoma; STAD, stomach adenocarcinoma. The asterisk indicates a significant difference of expression between a cancer type and the corresponding normal tissues (*p* < 0.01). Graphs were obtained from GEPIA2 at http://gepia2.cancer-pku.cn.

*RASSF1* isoforms expression is variable in different gastrointestinal cancer types (Figure 4).

**Figure 5:**
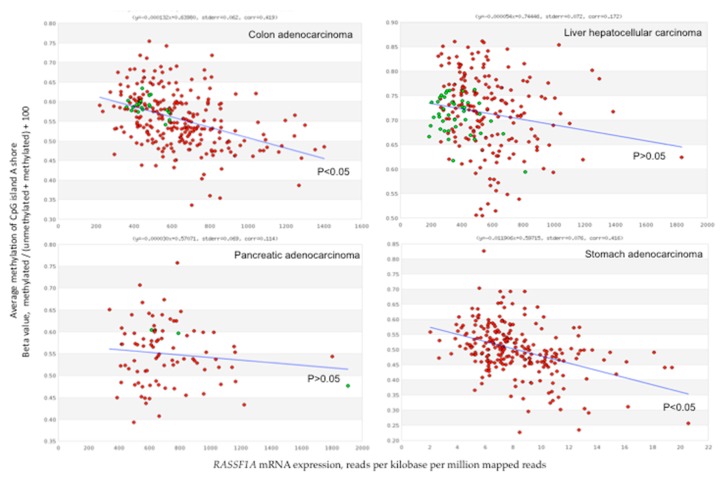
Average methylation of 15 CpGs in the CpG island A shore in function of the mRNA level of *RASSF1A* in colorectal, liver, pancreatic, and stomach cancers. Average methylation of cytosines in the CpG island A shore in function of the mRNA level (NM_007182) in colorectal, liver, pancreatic, and stomach cancers. *RASSF1A* mRNA expression represent the number of reads per kilobase per million mapped reads. CpG island A shore methylation correspond to the average methylation of 15 CpGs (chr3 from 50,378,611 to chr3:50,377,755, GRCh37/hg19). Red points, tumor tissues; green points, normal tissues. Blue line represents the trend line through the points. Parameters of the trend line, equation of the straight line, standard error, and correlation coefficient, are shown above graphs. A significant inverse correlation between CpG island A methylation and *RASSF1A* expression is found in colorectal and stomach cancers. The *p*-value of the trend line is indicated. The graphs, retrieved from the MethHC site [114], are based on The Cancer Genome Atlas (TCGA) data.

*RASSF1A* expression level is higher in stomach cancers and lower in liver cancers. *RASSF1C* level is, in general, higher than *RASSF1A* and *RASSF1B* level. *RASSF1C* expression is significantly higher in cancers than in normal tissues in cholangiocarcinoma and hepatocellular carcinoma (Figure 4). Certain studies have reported a robust correlation between 5mCs distribution and *RASSF1A* mRNA levels on one side, and methylation hotspots and transcription on the other. Concomitant *RASSF1A*m increase and gene expression loss has been reported in many studies regarding gastrointestinal cancers (Tables S1 to S5). However, TCGA data show no significant inverse correlation between promoter or 5′-UTR average DNA methylation and number of reads in colorectal, liver, pancreatic, and stomach cancers. High stringency is observed for methylation in the shore elements of the CpG island A in colorectal and stomach cancers (Figure 5).

Flanking regions of CpG islands, referred to as CpG island shores, showed tissue-specific DNA hypermethylation and association with gene silencing in cancer [61,120]. Based on TCGA data, CpG island A shore methylation correlated inversely with *RASSF1A* mRNA levels in colon and stomach cancers, but not in liver and pancreatic cancers (Figure 5). An average methylation of the whole *RASSF1A* promoter region higher than 20% results in a reduced *RASSF1A* mRNA expression in various cancer cell lines, suggesting that it represents a critical threshold for efficient gene silencing [109]. At this degree of methylation, it is probable that in each genome some of the regulatory CpGs of a gene are methylated, thereby establishing an efficient contrast to the transcription initiation complex.

## 8. Conclusions and Perspectives

Multiple lines of evidence demonstrate that loss of *RASSF1A* promotes cell transformation and that epigenetic regulation by DNA methylation may be one of the responsible mechanisms in a wide variety of malignancies. *RASSF1A*m is a widespread event in gastrointestinal cancers and promises to serve as a valuable diagnostic/prognostic marker, making it possible to translate epigenomics into clinical relevant information [121].

A large body of experimental data underline the importance of a controlled and adequate supply of *RASSF1A* for correct functionality of cells, whereas it is questionable if the current knowledge about the DNA methylation pattern is sufficient to allow the exploitation of DNA methylation data as a biomarker. Single CpGs may carry out specialized functions, in particular if they rule over the binding of transcription factors acting as master tissue homeostasis regulators [62]. Our ability to resolve unique patterns of methylation in complex arrays of different tissues is still limited and the use of different, non-comparable, techniques for the detection of methylation and relative expression of *RASSF1A* has counteracted its power as a reliable tumor marker and this limitation therefore encourages to adopt more standardized methods.

Although DNA methylation data have long been considered a promising source of biomarkers for cancer diagnosis, prognosis, and prediction, there are a few successful examples that confirmed the previous findings and were applied to clinics [122]. Concerning *RASSF1A*, its methylation was used a marker in a panel for the early detection of the hepatocellular carcinoma [90]. *RASSF1A*m was applied to clinics as tissue biomarker only for prostate cancer [123].

It is believed that a biomarker based on DNA methylation does not necessarily have to be correlated with gene expression. However, a correlation DNA methylation-gene expression provides a biological rationale to support the clinical application as biomarker of the DNA methylation. Koch et al. have used TCGA data on prostate cancer to assess the correlation between DNA methylation and mRNA expression of *RASSF1A* [122]. They showed that different assays aimed at determining *RASSF1A*m result in contradictory outcomes and insufficiently effective discriminating power (positive or negative or no correlation between *RASSF1A* methylation and mRNA expression). Analogous conclusions have been obtained for the methylation of other genes, suggesting that finding a reliable assay location is needed [122]. The conclusions drawn by Koch et al. challenge the results of previous studies on *RASSF1Am* and solicit a revision of available methods and strategies so far applied [122]. Well-designed/informative high resolution and quantitative DNA methylation and mRNA/protein analyses are required.

The exact location of biologically and clinically relevant hypermethylation of *RASSF1A*, with reference to specific contexts and pathologies, is still unknown. In addition to the promoter region, attention should be paid at distal enhancers. An association between deregulated gene expression and CpGs methylation in cancer may result significantly stronger for distal enhancers than the promoters of many genes [124]. To our knowledge, the role of the methylation of distal enhancer sequences in the *RASSF1A* expression regulation has never been established.

In conclusion, we do not know precisely why methylation of cytosines rises at a spot, if it originates from a random process, and/or if it is acquired through selection. In addition, the inhibitory efficiency of 5mCs at a certain CpG site on gene transcription is not easily predictable. The relevance of 5mCs at specific CpG positions might regard the loss of binding of transcription factors and of communication between signaling pathways and the functions powered by *RASSF1A*. Future DNA methylation analyses should extend assay locations, provide the patterns of methylation in single or few genomes complemented by the effect on the binding of transcription factors, and the consequent transcriptional output [125]. The exploitation of this knowledge is of strategic importance for the correct interpretation of the consequences that methylation plays on cellular function and to achieve robust associations with clinical data.

## Figures and Tables

**Table 1:** Frequency of *RASSF1A* methylation in gastrointestinal cancers *.

Cancer Type	Methylation of *RASSF1A*
Hepatocellular carcinoma (HCC)	522 of 669 (78%) HCC
Hepatoblastoma (HB)	46 of 133 (34.6%) HB
Esophageal squamous cell carcinoma (ESCC)	442 of 884 (50%) ESCC
Pancreatic ductal adenocarcinoma (PDAC)	32 of 59 (54%) PDAC
Pancreatic endocrine tumor (PET)	114 of 175 (75%) PET
Colorectal carcinoma (CRC)	558 of 1567 (35.6%) CRC
Gastric cancer (GC)	179 of 378 (31%) GC

* see Tables S1 to S5 for experimental details and references.

## Supplementary Materials

The following are available online at https://www.mdpi.com/2072-6694/11/7/959/s1, Table S1: *RASSF1A* methylation in liver neoplasms, Table S2: *RASSF1A* methylation in esophageal squamous cell carcinoma, Table S3: *RASSF1A* methylation in pancreatic neoplasms, Table S4: *RASSF1A* methylation in colorectal cancer, Table S5: *RASSF1A* methylation in gastric cancers.
