# Supplementary material for: Methylation Dynamics of RASSF1A and Its Impact on Cancer
Source: Cancers (Basel). 2019 Jul 9;11(7):959. doi: 10.3390/cancers11070959 (PMC6678546; doi:10.3390/cancers11070959)
Supplement: Supplementary file 1 [file cancers-11-00959-s001.pdf]

Supplementary Materials

Methylation Dynamics of *RASSF1A* and Its Impact on Cancer

Giorgio Malpeli, Giulio Innamorati, Ilaria Decimo, Maria Bencivenga, Armel Herve Nwabo Kamdje, Roberto Perris and Claudio Bassi

Table S1. *RASSF1A* methylation in liver neoplasms.

Cancer Type *	Rate of *RASSF1A* Methylation. Assay Location. Notes.	Detection Method	Association with Clinical Data	References
HCC	15 of 17 (88%) cancers. 2 of 7 (29%) cirrhotic tissues. Mean methylation level: 59% in 20 HCC, 26% in 9 cirrhotic, 16% in 12 non-cirrhotic tissues. 5 CpGs in first exon.	DNA pyrosequencing	YES	Araujo, O.C. et al., 2016 [1]
HCC	67 of 120 (85.5%) cancers. 48 of 120 (61.8%) normal cancer-adjacent liver. 9 of 35 (25.5%) hepatitis. 8 of 35 (23.6%) cirrhosis. 43 of 120 (36.3%) non liver tissues. Promoter, first exon.	Bisulfite DNA sequencing and quantitative MSP	n.d.	Jain, S. et al., 2015 [2]
HB	25 of 74 (34%) cancers. Promoter, first exon.	DNA pyrosequencing	YES	Honda, S. et al., 2013 [3]
HCC	82 of 103 (80%) cancers. Location unknown. *RASSF1A* methylation inversely correlated with mRNA and protein expression.	n.a.	NO	Feng, Y. et al., 2012 [4]
HCC	105 of 138 (76%) cancers. Mean methylation level: 53% in 138 HCC, 24% in 75 cirrhotic, 22% in 40 non-cirrhotic tissues, 10% in 28 healthy liver. 5 CpGs in first exon. Strong downregulation of *RASSF1A* mRNA expression compared to healthy liver.	DNA pyrosequencing	YES	Lambert, M.P. et al., 2011 [5]
HCC	31 of 35 (89%) cancers. Location unknown.	MSP	YES	Hu, L. et al., 2010 [6]
HCC	25 of 29 (86%) cancers. First exon. Loss of *RASSF1A* mRNA expression in 36% of HCC without correlation with methylation.	MSP	YES, better prognosis.	Saelee, P. et al., 2010 [7]
HB	6 of 20 (31%) cancers by MSP. 43 of 97 (44%) cancers by qMSP. Promoter, first exon.	MSP and quantitative MSP	NO by MSP.<br>YES by qMSP.	Honda, S. et al., 2008 [8]
HB	15 of 39 (39%) cancers. Promoter, first exon.	MSP and bisulfite DNA sequencing	YES	Sugawara, W. et al., 2007 [9]
GC	5 of 22 (27%) cancers. Promoter, first exon. Reduction or loss of *RASSF1A* expression was observed in most methylated GC.	MSP	n.d.	Kee, S.K. et al., 2007 [10]

GC	4 of 50 (8%) cancers. Promoter, first exon.	MSP	n.d.	Riquelme, E. et al., 2007 [11]
HCC	26 of 26 (100%) tissue. Promoter.	MSP	n.d.	Di Gioia, S. et al., 2006 [12]
HCC	6 of 20 (30%) cancers. First exon.	MSP	n.d.	Oliveira, C. et al., 2005 [13]
HCC	37 of 40 (93%) cancers. First exon. 17 of 40 (43%) in the plasma of the same patients.	MSP	n.d.	Yeo, W. et al., 2005 [14]
GC	10 of 37 (27%) cancers. First exon.	MSP	NO	Tozawa, T. et al., 2004 [15]
GC	0 of 50 (0%) cancers. Promoter, first exon.	MSP	n.d.	Takahashi, T. et al., 2004 [16]
HCC	41 of 43 (95%) in cancer. 16 of 23 (70%) in matched normal liver tissue. 16 CpGs in the promoter.	Bisulfite DNA sequencing	n.d.	Zhong, S. et al., 2003 [17]
HCC	71 of 83 (85%) cancers. Promoter.	MSP	n.d.	Zhang, Y.J. et al., 2002 [18]
HCC	14 of 15 (93%) cancers. 2 of 2 (100%) fibrosis. 3 of 4 (75%) cirrhosis. First exon.	COBRA	n.d.	Schagdarsurengin, U. et al., 2003 [19]
CC	9 of 13 (69%) cancers. First exon. Reduced *RASSF1A* mRNA expression in 9 CC cases.	MSP	n.d.	Wong, N. et al., 2002 [20]

* HCC, hepatocellular carcinoma; GC, gallbladder carcinoma; HB, hepatoblastoma; CC, cholangiocarcinoma; MSP, methylation-specific PCR; COBRA, combined bisulfite restriction analysis; n.a., not available; n.d., not determined.

Table S2. *RASSF1A* methylation in esophageal squamous cell carcinoma.

Cancer Type *	Rate of *RASSF1A* Methylation. Assay Location. Notes.	Detection Method	Association with Clinical Data	References
ESSC	35 of 71 (46.5%) cancers. 1 of 35 (2.9%) in normal cancer-adjacent mucosa. Lower *RASSF1A* expression in cancer than in normal.	MSP	YES	Guo, Q. et al., 2016 [21]
ESCC	45 of 100 (45%) cancers. First exon.	MSP	n.d.	Du, Z. et al., 2015 [22]
ESCC	78 of 141 (55%) cancers. 6 of 141 (4%) normal adjacent tissues. First exon. Decreased mRNA and protein expression of *RASSF1A* was observed in ESCC and was associated with promoter and exon methylation status.	MSP	YES	Guo, W. et al. 2014 [23]
ESCC	76 of 143 (53%) cancers. 10 of 62 (16%) normal adjacent cancers. First exon. No association between *RASSF1A* methylation and protein expression.	MSP	YES	Zhou, S.L. et al., 2013 [24]
ESCC	79 of 124 (64%) cancers. First exon. *RASSF1A* hypermethylation was statistically associated with loss of mRNA expression.	Quantitative MSP	YES	Mao, W.M. et al., 2011 [25]
ESCC	7 of 50 (17%) cancers. Promoter and first exon	MSP	n.d.	Kim, Y.T. et al., 2009 [26]
ESCC	32 of 66 (49%) cancers. Site unknown.	MSP	n.d.	Cong, D.G. and Wang, S.F., 2007 [27]
ESCC	22 of 64 (34%) cancers. Promoter and first exon	MSP	NO	Wong, M.L. et al., 2006 [28]
ESCC	3 of 55 (24%) cancers. Promoter and first exon	MSP	n.d.	Yamaguchi, S. et al., 2005 [29]
ESCC	11 of 22 (51%) cancers. Promoter and first exon.	MSP	n.d.	Kuroki, T. et al., 2003 [30]
ESCC	25 of 48 (52%) cancers. Promoter and first exon.	MSP	n.d.	Kuroki, T. et al., 2003 [31]

* ESCC, esophageal squamous cell carcinoma; MSP, methylation-specific PCR; n.d., not determined.

Table S3. *RASSF1A* methylation in pancreatic neoplasms.

Cancer Type *	Rate of *RASSF1A* Methylation. Assay Location. Notes.	Detection Method	Association with Clinical Data	References
PDAC	3 of 14 (26%) xenografts cancers. Promoter and first exon. *RASSF1A* mRNA expression always detected independently from methylation status. Protein expression lower in cancer than normal in 50% of PDAC.	DNA pyrosequencing	n.d.	Amato, E. et al., 2016 [32]
PET	16 of 20 (80%) cancers by MSP. 11 of 20 (55%) cancers by DNA pyrosequencing. Promoter, first exon. *RASSF1A* mRNA level was lower in PET than normal and correlated inversely with average methylation in PET and normal.	MSP and DNA pyrosequencing	n.d.	Malpeli, G. et al., 2011 [33]
PET	54–80% in specific subtypes.	MSP	n.d.	Arnold, C.N. et al., 2007 [34]
PET	10 of 16 (63%) cancers. First exon.	MSP	n.d.	Liu, L. et al., 2005 [35]
PET	10 of 10 (100%) cancers. First exon.	MSP	n.d.	Pizzi, S. et al., 2005 [36]
PDAC	29 of 45 (64%) cancers. First exon. *RASSF1A* silenced in metyhylated cases and cell lines.	MSP	n.d.	Dammann, R. et al., 2003 [37]
PET	10 of 12 (83%) cancers. First exon.	MSP	n.d.	Dammann, R. et al., 2003 [37]
PET	36 of 48 (75%) cancers. First exon.	MSP	YES	House, M.G. et al., 2003 [38]

* PDAC, pancreatic ductal adenocarcinoma; PET, pancreatic endocrine tumor; MSP, methylation-specific PCR; n.d., not determined.

Table S4. *RASSF1A* methylation in colorectal cancer.

Cancer Type *	Rate of *RASSF1A* Methylation. Assay Location. Notes.	Detection Method	Association with Clinical Data	References
CRC	Higher methylation level in 100 CRC tissues compared to normal adjacent mucosa. Promoter.	DNA pyrosequencing	n.d.	Sugai, T. et al., 2017 [39]
CRC	18 of 51 (35%) cancers. 43 of 51 (85%) of paired liver metastases. Site unknown.	Quantitative MSP	n.d.	Schirosi, L. et al., 2016 [40]
CRC	42 of 65 (65%) cancers. 10 of 65 (15%) normal mucosa. Promoter.	MSP	YES	Li, M. et al., 2016 [41]
CRC	10 of 80 (12%) cancers. 1 of 80 (1%) adjacent normal mucosa. First exon.	Methylation-sensitive high resolution melting	YES	Coppede, F. et al., 2014 [42]
CRC	29 of 62 (47%) cancers. Promoter, first exon.	MSP	YES	Sinha, R. et al., 2013 [43]
CRC	16 of 111 (14%) cancers, average methylation 31%. Normal mucosa never methylated. Promoter, first exon.	DNA pyrosequencing	YES	Nilsson, T.K. et al., 2013 [44]
CRC	86 of 117 (74%) cancers. Site unknown.	n.a.	NO	Chen, S.P. et al., 2012 [45]
CRC	20 of 100 (20%) cancers. 9% in normal adjacent tumor cancers having verage methylation > 10%. First exon.	Quantitative PCR		Kang, H.J. et al., 2012 [46]
CRC	17 of 36 (47%) cancers. First exon.	MSP	NO	Abouzeid, H.E. et al., 2011 [47]
CRC	26 of 94 (28%) cancers. Methylation >15% in 22 CpGs. Normal mucosa: average methylation 18% (5-34%). No difference at 2 cm and 10 cm from cancer. Average methylation higher in distal than proximal normal mucosa. First exon.	DNA pyrosequencing and bisulfite DNA sequencing	NO	An, B. et al., 2010 [48]
CRC	8 of 47 (17%) cancers. 1 of 47 (2%) normal mucosa. First exon.	Quantitative PCR	YES	Gonzalo, V. et al., 2010 [49]
CRC	26 of 73 (36%) cancers. 14% in paired normal tissues. First exon.	MSP	NO	Miladi-Abdennadher, I. et al., 2010 [50]
CRC	10 of 12 (83%) cancers. 9 of 13 (69%) of normal colonic mucosa. Promoter.	MSP	n.d.	Minoo, P. et al., 2006 [51]
CRC	6 of 25 (24%) cancers. First exon. *RASSF1A* mRNA expression lower in methylated cases.	MSP	n.d.	Greenspan, E.J. et al., 2006 [52]
CRC	40 of 202 (20%) cancers. First exon.	MSP	n.d.	Miranda, E. et al., 2006 [53]
CRC	16 of 31 (52%) sporadic MSI cancers. First exon.	MSP	n.d.	Oliveira, C. et al., 2005 [13]
CRC	12 of 28 (43%) cancers. First exon.	MSP	n.d.	Dong, S.M. et al., 2005 [54]
CRC	39 of 48 (81%) cancers. 19 of 39 (49%) normal colonic mucosa. First exon.	MSP	n.d.	Sakamoto, N. et al., 2004 [55]
CRC	2 of 65 (3%) cancers. First exon.	MSP	n.d.	Xu, X.L. et al., 2004 [56]
CRC	45 of 220 (20%) cancers. First exon.	MSP	n.d.	van Engeland, M. et al., 2002 [57]

* CRC, colorectal carcinoma; MSP, methylation-specific PCR; n.a., not available; n.d., not determined.

Table S5. *RASSF1A* methylation in gastric cancers.

Cancer Type *	Rate of *RASSF1A* Methylation. Assay Location. Notes.	Detection Method	Association with Clinical Data	References
GC	14 of 21 (67%) cancers and 9 of 26 (35%) normal adjacent cancers (with metaplasia). First exon. *RASSF1A* expression was significantly reduced in methylated cases.	MSP	n.d.	Joo, M.K. et al., 2015 [58]
GC	13 of 102 (13%) cancers. First exon.	MSP	NO	Li, Y. et al., 2015 [59]
GCA	60 of 92 (65%) cancers. 6 of 30 (20%) normal adjacent cancer. First exon.	MSP	YES	Zhou, S.L. et al., 2013 [24]
GC	0 of 98 (0%) cancers. Average methylation 4.3% in 6 CpGs. First exon.	DNA pyrosequencing	NO	Balassiano, K. et al., 2011 [60]
GCA	54 of 92 (59%) cancers. Methylation in cancer higher than normal tissues. Promoter. Reduced *RASSF1A* mRNA and protein expression in cancer compared to normal tissues.	MSP	n.d.	Guo, W. et al., 2009 [61]
GC	0 of 16 (0%) cancers. No methylation in normal, metaplasia and displasia. First exon. No reduction of *RASSF1A* protein expression was observed in cancer compared to normal.	MSP	NO	Zou, X.P. et al., 2009 [62]
GC	36 of 54 (67%) cancers. *RASSF1A* methylation was higher in cancer than in corresponding normal tissues (66.7% vs. 14.8%). Promoter, first exon. *RASSF1A* mRNA and protein expression was reduced in cancer compared to normal tissues.	MSP	n.d.	Ye, M. et al., 2007 [63]
GC	11 of 25 (44%) cancers. First exon.	MSP	n.d.	Oliveira, C. et al., 2005 [13]
GC	6 of 80 (7.5%) cancers. Never found in adenomas, metaplasia, gastritis. First exon.	MSP	n.d.	Kang, G.H. et al., 2003 [64]
GC	39 of 90 (44%) cancers. 0 of 15 (0%) normal tissues. Promoter. Low level of *RASSF1A* mRNA in 46% and complete loss in 13% of 90 GC. No *RASSF1A* expression in cases and cell lines with LOH.	MSP, bisulfite DNA sequencing.	YES	Byun, D.S. et al., 2001 [65]

* GC, gastric carcinoma; GCA, gastric cardia adenocarcinoma; MSP, methylation-specific PCR; n.d., not determined.

References

1. Araujo, O.C.; Rosa, A.S.; Fernandes, A.; Niel, C.; Villela-Nogueira, C.A.; Pannain, V.; Araujo, N.M. RASSF1A and DOK1 Promoter Methylation Levels in Hepatocellular Carcinoma, Cirrhotic and Non-Cirrhotic Liver, and Correlation with Liver Cancer in Brazilian Patients. *PLoS ONE* **2016,** *11*, e0153796.
2. Jain, S.; Xie, L.; Boldbaatar, B.; Lin, S.Y.; Hamilton, J.P.; Meltzer, S.J.; Chen, S.H.; Hu, C.T.; Block, T.M.; Song, W.; et al. Differential methylation of the promoter and first exon of the RASSF1A gene in hepatocarcinogenesis. *Hepatol. Res.* **2015,** *45*, 1110–1123.
3. Honda, S.; Miyagi, H.; Suzuki, H.; Minato, M.; Haruta, M.; Kaneko, Y.; Hatanaka, K.C.; Hiyama, E.; Kamijo, T.; Okada, T.; et al. RASSF1A methylation indicates a poor prognosis in hepatoblastoma patients. *Pediatr. Surg. Int.* **2013,** *29*, 1147–1152.
4. Feng, Y.; Xue, W.J.; Li, P.; Sha, Z.Y.; Huang, H.; Rui, L.; Li, H.X.; Mao, Q.S. RASSF1A hypermethylation is associated with aflatoxin B1 and polycyclic aromatic hydrocarbon exposure in hepatocellular carcinoma. *Hepatogastroenterology* **2012,** *59*, 1883–1888.
5. Lambert, M.P.; Paliwal, A.; Vaissiere, T.; Chemin, I.; Zoulim, F.; Tommasino, M.; Hainaut, P.; Sylla, B.; Scoazec, J.Y.; Tost, J.; et al. Aberrant DNA methylation distinguishes hepatocellular carcinoma associated with HBV and HCV infection and alcohol intake. *J. Hepatol.* **2011,** *54*, 705–715.
6. Hu, L.; Chen, G.; Yu, H.; Qiu, X. Clinicopathological significance of RASSF1A reduced expression and hypermethylation in hepatocellular carcinoma. *Hepatol. Int.* **2010,** *4*, 423–432.
7. Saelee, P.; Wongkham, S.; Chariyalertsak, S.; Petmitr, S.; Chuensumran, U. RASSF1A promoter hypermethylation as a prognostic marker for hepatocellular carcinoma. *Asian Pac. J. Cancer Prev.* **2010,** *11*, 1677–1681.
8. Honda, S.; Haruta, M.; Sugawara, W.; Sasaki, F.; Ohira, M.; Matsunaga, T.; Yamaoka, H.; Horie, H.; Ohnuma, N.; Nakagawara, A.; et al. The methylation status of RASSF1A promoter predicts responsiveness to chemotherapy and eventual cure in hepatoblastoma patients. *Int. J. Cancer* **2008,** *123*, 1117–1125.
9. Sugawara, W.; Haruta, M.; Sasaki, F.; Watanabe, N.; Tsunematsu, Y.; Kikuta, A.; Kaneko, Y. Promoter hypermethylation of the RASSF1A gene predicts the poor outcome of patients with hepatoblastoma. *Pediatr. Blood Cancer* **2007,** *49*, 240–249.
10. Kee, S.K.; Lee, J.Y.; Kim, M.J.; Lee, S.M.; Jung, Y.W.; Kim, Y.J.; Park, J.Y.; Bae, H.I.; Hong, H.S.; Yun, Y.K.; et al. Hypermethylation of the Ras association domain family 1A (RASSF1A) gene in gallbladder cancer. *Mol. Cells* **2007,** *24*, 364–371.
11. Riquelme, E.; Tang, M.; Baez, S.; Diaz, A.; Pruyas, M.; Wistuba, I.I.; Corvalan, A. Frequent epigenetic inactivation of chromosome 3p candidate tumor suppressor genes in gallbladder carcinoma. *Cancer Lett.* **2007,** *250*, 100–106.
12. Di Gioia, S.; Bianchi, P.; Destro, A.; Grizzi, F.; Malesci, A.; Laghi, L.; Levrero, M.; Morabito, A.; Roncalli, M. Quantitative evaluation of RASSF1A methylation in the non-lesional, regenerative and neoplastic liver. *BMC Cancer* **2006**, *6*, 89.
13. Oliveira, C.; Velho, S.; Domingo, E.; Preto, A.; Hofstra, R.M.; Hamelin, R.; Yamamoto, H.; Seruca, R.; Schwartz, S., Jr. Concomitant RASSF1A hypermethylation and KRAS/BRAF mutations occur preferentially in MSI sporadic colorectal cancer. *Oncogene* **2005,** *24*, 7630–7634.
14. Yeo, W.; Wong, N.; Wong, W.L.; Lai, P.B.; Zhong, S.; Johnson, P.J. High frequency of promoter hypermethylation of RASSF1A in tumor and plasma of patients with hepatocellular carcinoma. *Liver Int.* **2005,** *25*, 266–272.
15. Tozawa, T.; Tamura, G.; Honda, T.; Nawata, S.; Kimura, W.; Makino, N.; Kawata, S.; Sugai, T.; Suto, T.; Motoyama, T. Promoter hypermethylation of DAP-kinase is associated with poor survival in primary biliary tract carcinoma patients. *Cancer Sci.* **2004,** *95*, 736–740.
16. Takahashi, T.; Shivapurkar, N.; Riquelme, E.; Shigematsu, H.; Reddy, J.; Suzuki, M.; Miyajima, K.; Zhou, X.; Bekele, B.N.; Gazdar, A.F.; Wistuba, I.I. Aberrant promoter hypermethylation of multiple genes in gallbladder carcinoma and chronic cholecystitis. *Clin. Cancer Res.* **2004,** *10*, 6126–6133.
17. Zhong, S.; Yeo, W.; Tang, M.W.; Wong, N.; Lai, P.B.; Johnson, P.J. Intensive hypermethylation of the CpG island of Ras association domain family 1A in hepatitis B virus-associated hepatocellular carcinomas. *Clin. Cancer Res.* **2003,** *9*, 3376–3382.
18. Zhang, Y. J.; Ahsan, H.; Chen, Y.; Lunn, R. M.; Wang, L. Y.; Chen, S. Y.; Lee, P. H.; Chen, C. J.; Santella, R. M. High frequency of promoter hypermethylation of RASSF1A and p16 and its relationship to aflatoxin B1-DNA adduct levels in human hepatocellular carcinoma. *Mol. Carcinog.* **2002,** *35*, 85-92.

19. Schagdarsurengin, U.; Wilkens, L.; Steinemann, D.; Flemming, P.; Kreipe, H. H.; Pfeifer, G. P.; Schlegelberger, B.; Dammann, R. Frequent epigenetic inactivation of the RASSF1A gene in hepatocellular carcinoma. *Oncogene* **2003,** *22*, 1866–1871.
20. Wong, N.; Li, L.; Tsang, K.; Lai, P.B.; To, K.F.; Johnson, P.J. Frequent loss of chromosome 3p and hypermethylation of RASSF1A in cholangiocarcinoma. *J. Hepatol.* **2002,** *37*, 633–639.
21. Guo, Q.; Wang, H.B.; Li, Y.H.; Li, H.F.; Li, T.T.; Zhang, W.X.; Xiang, S.S.; Sun, Z.Q. Correlations of Promoter Methylation in WIF-1, RASSF1A, and CDH13 Genes with the Risk and Prognosis of Esophageal Cancer. *Med. Sci. Monit* **2016**, *22*, 2816–2824.
22. Du, Z.; Ma, K.; Sun, X.; Li, A.; Wang, H.; Zhang, L.; Lin, F.; Feng, X.; Song, J. Methylation of RASSF1A gene promoter and the correlation with DNMT1 expression that may contribute to esophageal squamous cell carcinoma. *World J. Surg. Oncol.* **2015**, *13*, 141.
23. Guo, W.; Cui, L.; Wang, C.; Guo, Y.; Shen, S.; Kuang, G.; Dong, Z. Decreased expression of RASSF1A and up-regulation of RASSF1C is associated with esophageal squamous cell carcinoma. *Clin. Exp. Metastasis* **2014,** *31*, 521–533.
24. Zhou, S.L.; Cui, J.; Fan, Z.M.; Li, X.M.; Li, J.L.; Liu, B.C.; Zhang, D.Y.; Liu, H.Y.; Zhao, X.K.; Song, X.; et al. Polymorphism of A133S and promoter hypermethylation in Ras association domain family 1A gene (RASSF1A) is associated with risk of esophageal and gastric cardia cancers in Chinese population from high incidence area in northern China. *BMC Cancer* **2013**, *13*, 259.
25. Mao, W.M.; Li, P.; Zheng, Q.Q.; Wang, C.C.; Ge, M.H.; Hu, F.J.; Fang, X.H.; Dong, Z.M.; Ling, Z.Q. Hypermethylation-modulated downregulation of RASSF1A expression is associated with the progression of esophageal cancer. *Arch Med. Res.* **2011,** *42*, 182–188.
26. Kim, Y.T.; Park, J.Y.; Jeon, Y.K.; Park, S.J.; Song, J.Y.; Kang, C.H.; Sung, S.W.; Kim, J.H. Aberrant promoter CpG island hypermethylation of the adenomatosis polyposis coli gene can serve as a good prognostic factor by affecting lymph node metastasis in squamous cell carcinoma of the esophagus. *Dis. Esophagus.* **2009,** *22*, 143–150.
27. Cong, D.G.; Wang, S.F. Hypermethylation of promoter region of RAS association domain family gene1A in esophageal squamous cell carcinoma and significance thereof. *Zhonghua Yi Xue Za Zhi* **2007,** *87*, 2932–2934.
28. Wong, M.L.; Tao, Q.; Fu, L.; Wong, K.Y.; Qiu, G.H.; Law, F.B.; Tin, P.C.; Cheung, W.L.; Lee, P.Y.; Tang, J.C.; et al. Aberrant promoter hypermethylation and silencing of the critical 3p21 tumour suppressor gene, RASSF1A, in Chinese oesophageal squamous cell carcinoma. *Int. J. Oncol.* **2006,** *28*, 767–773.
29. Yamaguchi, S.; Kato, H.; Miyazaki, T.; Sohda, M.; Kimura, H.; Ide, M.; Asao, T.; Kuwano, H. RASSF1A gene promoter methylation in esophageal cancer specimens. *Dis. Esophagus.* **2005,** *18*, 253–256.
30. Kuroki, T.; Trapasso, F.; Yendamuri, S.; Matsuyama, A.; Alder, H.; Mori, M.; Croce, C.M. Allele loss and promoter hypermethylation of VHL, RAR-beta, RASSF1A, and FHIT tumor suppressor genes on chromosome 3p in esophageal squamous cell carcinoma. *Cancer Res.* **2003,** *63*, 3724–3728.
31. Kuroki, T.; Trapasso, F.; Yendamuri, S.; Matsuyama, A.; Alder, H.; Mori, M.; Croce, C.M. Promoter hypermethylation of RASSF1A in esophageal squamous cell carcinoma. *Clin Cancer Res.* **2003,** *9*, 1441–1445.
32. Amato, E.; Barbi, S.; Fassan, M.; Luchini, C.; Vicentini, C.; Brunelli, M.; Malleo, G.; Scarpa, A.; Malpeli, G. RASSF1 tumor suppressor gene in pancreatic ductal adenocarcinoma: Correlation of expression, chromosomal status and epigenetic changes. *BMC Cancer* **2016,** *16*, 11.
33. Malpeli, G.; Amato, E.; Dandrea, M.; Fumagalli, C.; Debattisti, V.; Boninsegna, L.; Pelosi, G.; Falconi, M.; Scarpa, A. Methylation-associated down-regulation of RASSF1A and up-regulation of RASSF1C in pancreatic endocrine tumors. *BMC Cancer* **2011**, *11*, 351.
34. Arnold, C. N.; Sosnowski, A.; Schmitt-Graff, A.; Arnold, R.; Blum, H. E. Analysis of molecular pathways in sporadic neuroendocrine tumors of the gastro-entero-pancreatic system. *Int. J. Cancer* **2007,** *120*, 2157–2164.
35. Liu, L.; Zhang, J.; Bates, S.; Li, J.J.; Peehl, D.M.; Rhim, J.S.; Pfeifer, G.P. A methylation profile of in vitro immortalized human cell lines. *Int J Oncol* **2005,** *26*, 275–285.
36. Pizzi, S.; Azzoni, C.; Bottarelli, L.; Campanini, N.; D'Adda, T.; Pasquali, C.; Rossi, G.; Rindi, G.; Bordi, C. RASSF1A promoter methylation and 3p21.3 loss of heterozygosity are features of foregut, but not midgut and hindgut, malignant endocrine tumours. *J. Pathol.* **2005,** *206*, 409–416.
37. Dammann, R.; Schagdarsurengin, U.; Liu, L.; Otto, N.; Gimm, O.; Dralle, H.; Boehm, B.O.; Pfeifer, G.P.; Hoang-Vu, C. Frequent RASSF1A promoter hypermethylation and K-ras mutations in pancreatic carcinoma. *Oncogene* **2003,** *22*, 3806–3812.

38. House, M.G.; Guo, M.; Efron, D.T.; Lillemoe, K.D.; Cameron, J.L.; Syphard, J.E.; Hooker, C.M.; Abraham, S.C.; Montgomery, E.A.; Herman, J.G.; et al. Tumor suppressor gene hypermethylation as a predictor of gastric stromal tumor behavior. *J. Gastrointest. Surg.* **2003,** *7*, 1004–1014.
39. Sugai, T.; Yoshida, M.; Eizuka, M.; Uesugii, N.; Habano, W.; Otsuka, K.; Sasaki, A.; Yamamoto, E.; Matsumoto, T.; Suzuki, H. Analysis of the DNA methylation level of cancer-related genes in colorectal cancer and the surrounding normal mucosa. *Clin Epigenetics* **2017**, *9*, 55.
40. Schirosi, L.; Mazzotta, A.; Opinto, G.; Pinto, R.; Graziano, G.; Tommasi, S.; Fucci, L.; Simone, G.; Mangia, A. beta-catenin interaction with NHERF1 and RASSF1A methylation in metastatic colorectal cancer patients. *Oncotarget* **2016,** *7*, 67841–67850.
41. Li, M.; Yan, D.G.; Liu, J.L. Methylation status of PCDH10 and RASSF1A gene promoters in colorectal cancer. *Zhonghua Yi Xue Za Zhi* **2016,** *96*, 456–459.
42. Coppede, F.; Migheli, F.; Lopomo, A.; Failli, A.; Legitimo, A.; Consolini, R.; Fontanini, G.; Sensi, E.; Servadio, A.; Seccia, M.; et al. Gene promoter methylation in colorectal cancer and healthy adjacent mucosa specimens: Correlation with physiological and pathological characteristics, and with biomarkers of one-carbon metabolism. *Epigenetics* **2014,** *9*, 621–633.
43. Sinha, R.; Hussain, S.; Mehrotra, R.; Kumar, R.S.; Kumar, K.; Pande, P.; Doval, D.C.; Basir, S.F.; Bharadwaj, M. Kras gene mutation and RASSF1A, FHIT and MGMT gene promoter hypermethylation: Indicators of tumor staging and metastasis in adenocarcinomatous sporadic colorectal cancer in Indian population. *PLoS ONE* **2013,** *8*, e60142.
44. Nilsson, T.K.; Lof-Ohlin, Z.M.; Sun, X.F. DNA methylation of the p14ARF, RASSF1A and APC1A genes as an independent prognostic factor in colorectal cancer patients. *Int J Oncol* **2013,** *42*, 127–133.
45. Chen, S.P.; Wu, C.C.; Huang, S.Y.; Kang, J.C.; Chiu, S.C.; Yang, K.L.; Pang, C.Y. beta-catenin and K-ras mutations and RASSF1A promoter methylation in Taiwanese colorectal cancer patients. *Genet Test Mol. Biomarkers* **2012,** *16*, 1277–1281.
46. Kang, H.J.; Kim, E.J.; Kim, B.G.; You, C.H.; Lee, S.Y.; Kim, D.I.; Hong, Y.S. Quantitative analysis of cancer-associated gene methylation connected to risk factors in Korean colorectal cancer patients. *J. Prev. Med. Public Health* **2012,** *45*, 251–258.
47. Abouzeid, H.E.; Kassem, A.M.; Abdel Wahab, A.H.; El-mezayen, H.A.; Sharad, H.; Abdel Rahman, S. Promoter hypermethylation of RASSF1A, MGMT, and HIC-1 genes in benign and malignant colorectal tumors. *Tumour Biol.* **2011,** *32*, 845–852.
48. An, B.; Kondo, Y.; Okamoto, Y.; Shinjo, K.; Kanemitsu, Y.; Komori, K.; Hirai, T.; Sawaki, A.; Tajika, M.; Nakamura, T.; et al. Characteristic methylation profile in CpG island methylator phenotype-negative distal colorectal cancers. *Int J Cancer* **2010,** *127*, 2095–2105.
49. Gonzalo, V.; Lozano, J.J.; Munoz, J.; Balaguer, F.; Pellise, M.; Rodriguez de Miguel, C.; Andreu, M.; Jover, R.; Llor, X.; Giraldez, M.D.; et al. Aberrant gene promoter methylation associated with sporadic multiple colorectal cancer. *PLoS ONE* **2010,** *5*, e8777.
50. Miladi-Abdennadher, I.; Abdelmaksoud-Damak, R.; Ayadi, L.; Khabir, A.; Frikha, F.; Kallel, L.; Amouri, A.; Frikha, M.; Sellami-Boudawara, T.; Gargouri, A.; et al. Hypermethylation of RARbeta2 correlates with high COX-2 expression and poor prognosis in patients with colorectal carcinoma. *Tumour Biol.* **2010,** *31*, 503–511.
51. Minoo, P.; Baker, K.; Goswami, R.; Chong, G.; Foulkes, W.D.; Ruszkiewicz, A.R.; Barker, M.; Buchanan, D.; Young, J.; Jass, J.R. Extensive DNA methylation in normal colorectal mucosa in hyperplastic polyposis. *Gut* **2006,** *55*, 1467–1474.
52. Greenspan, E.J.; Jablonski, M.A.; Rajan, T.V.; Levine, J.; Belinsky, G.S.; Rosenberg, D.W. Epigenetic alterations in RASSF1A in human aberrant crypt foci. *Carcinogenesis* **2006,** *27*, 1316–1322.
53. Miranda, E.; Destro, A.; Malesci, A.; Balladore, E.; Bianchi, P.; Baryshnikova, E.; Franchi, G.; Morenghi, E.; Laghi, L.; Gennari, L.; et al. Genetic and epigenetic changes in primary metastatic and nonmetastatic colorectal cancer. *Br. J. Cancer* **2006,** *95*, 1101–1107.
54. Dong, S.M.; Lee, E.J.; Jeon, E.S.; Park, C.K.; Kim, K.M. Progressive methylation during the serrated neoplasia pathway of the colorectum. *Mod. Pathol.* **2005,** *18*, 170–178.
55. Sakamoto, N.; Terai, T.; Ajioka, Y.; Abe, S.; Kobayasi, O.; Hirai, S.; Hino, O.; Watanabe, H.; Sato, N.; Shimoda, T.; et al. Frequent hypermethylation of RASSF1A in early flat-type colorectal tumors. *Oncogene* **2004,** *23*, 8900–8907.

56. Xu, X.L.; Yu, J.; Zhang, H.Y.; Sun, M.H.; Gu, J.; Du, X.; Shi, D.R.; Wang, P.; Yang, Z.H.; Zhu, J.D. Methylation profile of the promoter CpG islands of 31 genes that may contribute to colorectal carcinogenesis. *World J. Gastroenterol.* **2004,** *10*, 3441–3454.
57. van Engeland, M.; Roemen, G.M.; Brink, M.; Pachen, M.M.; Weijenberg, M.P.; de Bruine, A.P.; Arends, J.W.; van den Brandt, P.A.; de Goeij, A.F.; Herman, J.G. K-ras mutations and RASSF1A promoter methylation in colorectal cancer. *Oncogene* **2002,** *21*, 3792–3795.
58. Joo, M.K.; Kim, K.H.; Park, J.J.; Yoo, H.S.; Choe, J.; Kim, H.J.; Lee, B.J.; Kim, J.S.; Bak, Y.T. CpG island promoter hypermethylation of Ras association domain family 1A gene contributes to gastric carcinogenesis. *Mol. Med. Rep.* **2015,** *11*, 3039–3046.
59. Li, Y.; Yang, Y.; Lu, Y.; Herman, J.G.; Brock, M.V.; Zhao, P.; Guo, M. Predictive value of CHFR and MLH1 methylation in human gastric cancer. *Gastric Cancer* **2015,** *18*, 280–287.
60. Balassiano, K.; Lima, S.; Jenab, M.; Overvad, K.; Tjonneland, A.; Boutron-Ruault, M.C.; Clavel-Chapelon, F.; Canzian, F.; Kaaks, R.; Boeing, H.; et al. Aberrant DNA methylation of cancer-associated genes in gastric cancer in the European Prospective Investigation into Cancer and Nutrition (EPIC-EURGAST). *Cancer Lett.* **2011,** *311*, 85–95.
61. Guo, W.; Dong, Z.; Chen, Z.; Yang, Z.; Wen, D.; Kuang, G.; Guo, Y.; Shan, B. Aberrant CpG island hypermethylation of RASSF1A in gastric cardia adenocarcinoma. *Cancer Invest.* **2009,** *27*, 459–465.
62. Zou, X.P.; Zhang, B.; Zhang, X.Q.; Chen, M.; Cao, J.; Liu, W.J. Promoter hypermethylation of multiple genes in early gastric adenocarcinoma and precancerous lesions. *Hum. Pathol.* **2009,** *40*, 1534–1542.
63. Ye, M.; Xia, B.; Guo, Q.; Zhou, F.; Zhang, X. Association of diminished expression of RASSF1A with promoter methylation in primary gastric cancer from patients of central China. *BMC Cancer* **2007**, *7*, 120.
64. Kang, G.H.; Lee, S.; Kim, J.S.; Jung, H.Y. Profile of aberrant CpG island methylation along the multistep pathway of gastric carcinogenesis. *Lab. Invest.* **2003,** *83*, 635–641.
65. Byun, D.S.; Lee, M.G.; Chae, K.S.; Ryu, B.G.; Chi, S.G. Frequent epigenetic inactivation of RASSF1A by aberrant promoter hypermethylation in human gastric adenocarcinoma. *Cancer Res.* **2001,** *61*, 7034–7038.

© 2019 by the authors. Licensee MDPI, Basel, Switzerland. This article is an open access article distributed under the terms and conditions of the Creative Commons Attribution (CC BY) license (http://creativecommons.org/licenses/by/4.0/).
